# Serum cholesterol level as a predictive biomarker for prognosis of Neuroblastoma

**DOI:** 10.1186/s12887-024-04700-7

**Published:** 2024-03-22

**Authors:** Jie Min, Yi Wu, Shungen Huang, Yanhong Li, Xinjing Lv, Ruze Tang, He Zhao, Jian Wang

**Affiliations:** Children’s Hospital of Soochow University, Pediatric Research Institute of Soochow University, Suzhou, 215123 Jiangsu China

**Keywords:** Neuroblastoma, Serum cholesterol, Prognosis, Predictive biomarker

## Abstract

**Background:**

Neuroblastoma (NB), a type of solid tumor in children, has a poor prognosis. Few blood biomarkers can accurately predict the prognosis, including recurrence and survival, in children with NB. In this study, we found that the serum total cholesterol (Tchol) level was associated with the prognosis of patients through a retrospective study.

**Methods:**

Multivariate Cox regression model was used to identify the independent risk factors in the children with NB. Kaplan–Meier method was used to analyze the correlation between the common biomarkers, including the serum Tchol level, and the prognosis of the patients. ROC curves were used to predict the accuracy of the International Neuroblastoma Staging System (INSS) stage and Children’s Oncology Group (COG) risk stratification after adding the serum Tchol level.

**Results:**

Compared with the other patients, serum Tchol level was significantly increased in the relapsed and died patients (*P* < 0.05). Subsequently, serum Tchol level was found as an independent risk factor to affect the outcome of patients (*P* < 0.05). Finally, we added serum Tchol level into traditional stage and risk classification system to form the new INSS stage and COG risk classification system. It was found that the areas under the ROC curve (AUC) of recurrence-free survival in the new INSS stage and COG risk classification system were increased to 0.691 (95%CI: 0.535–0.847) and 0.748 (95%CI: 0.622–0.874), respectively. Moreover, the AUC areas of overall survival in the new INSS stage and COG risk classification system were increased to 0.722 (95%CI: 0.561–0.883) and 0.668 (95%CI: 0.496–0.819), respectively.

**Conclusion:**

We found that serum Tchol level, a clinical biomarker, is a risk factor for recurrence and death among the children with NB. The serum Tchol level could significantly increase the accuracy of the prediction for NB prognosis.

**Supplementary Information:**

The online version contains supplementary material available at 10.1186/s12887-024-04700-7.

## Introduction

Neuroblastoma (NB) is the most common extracranial solid tumor in childhood, accounting for approximately 8% of all childhood malignancies and 15% of childhood tumor mortality [1]. The clinicians use the International Neuroblastoma Staging System (INSS) stage and the Children’s Oncology Group (COG) risk stratification systems to analyze, group and treat children with NB. Nevertheless, about 50% to 60% of patients in the high-risk group are likely to relapse [2]. The traditional COG risk classification system is commonly used to predict prognosis in clinic. However, this system, a kind of prognostic evaluation system based on multiple factors, is more complicated than a single biomarker. On the other hand, common biomarkers, including neuron-specific enolase (NSE), vanillylmandelic acid and homovanillic acid, offer low sensitivity to predict the progression of NB [3]. Therefore, there is a significant demand to find a simple and effective biomarker to accurately predict the prognosis of the patients [4].

Cholesterol, a biochemistry index in serum to maintain normal physiological functions, is a kind of sterol that can be easily measured in clinic. More and more evidence has shown that cholesterol may play an important role in the occurrence and progression of tumors [5–8]. High levels of cholesterol can promote the rapid proliferation of cancer cells and affect the prognosis of patients with endometrial cancer [9] and breast cancer [10]. The drugs with lowering cholesterol levels include statins, cholesterol absorption inhibitors (Ezetimibe), antioxidants (Probucol), and bile acid binding resins (Colestyramine). It has been reported that statins have anti-proliferative, pro-apoptotic and anti-invasive effects on tumor cells by decreasing the level of cholesterol [11]. However, it is still unclear that the role of serum cholesterol in the progression of NB. Therefore, the objective of the study was to examine serum cholesterol's potential as a predictive biomarker for NB and the correlation between the level of serum Tchol and the prognosis of the patients.

This study began with a retrospective analysis in patients with NB. Based on the data of recurrence-free survival (RFS) and overall survival (OS), the correlation between the level of serum cholesterol and the prognosis of the children with NB was then studied in detail. Moreover, the level of serum cholesterol, a potential biomarker, was combined with the other markers to enhance the accuracy of predicting the prognosis of NB. Therefore, this work not only provides a simple and accurate biomarker and method for prognosis prediction of children with NB, but also may provide a direction in therapeutic strategy of NB.

## Method

### General information

In this study, we selected NB cases that were diagnosed by bone marrow (BM) examination or surgery in the Children's Hospital of Soochow University from 2013 to 2021. BM cell morphology and pathology were diagnosed by two or more senior specialists. The representative immunohistochemistry (IHC) images of NB with different pathological diagnostic markers were provided by the Children's Hospital of Soochow University (Supplementary Fig. 1). The family of the patient was followed up by telephone or in the outpatient service of the hospital.

Inclusion criteria were as follows: (1) pathological diagnosis of NB, (2) BM examination for characteristics of NB (small round cells arranged in nests or chrysanthemum clumps) along with elevated serum levels of NSE or catecholamine metabolites, (3) cases with complete clinical data except for MYCN status and serum NSE level. Exclusion criteria were as follows: (1) cases who did not choose further treatment, (2) cases that were lost during follow-up, (3) cases that showed new lesions during chemotherapy.

### Definition of relevant research variables

All data is from the electronic medical record system of the Children's Hospital of Soochow University. The study has general demographics, including age, gender, ethnicity, BMI (Body Mass Index), imaging-related data (blood vessels around the tumor based on image-defined risk factors, primary site and metastasis sites), laboratory variables (the levels of serum NSE, lactate dehydrogenase (LDH) and Tchol before any treatment, MYCN status in BM detected by fluorescence in situ hybridization), tumor-related data (pathological type based on International Neuroblastoma Pathology Classification, INSS stage and COG risk classification system). The images of some children cannot distinguish whether the tumor originated from the adrenal gland or the retroperitoneum. Therefore, these groups of children were combined for further analysis in this study. According to the grouped prognostic classification, the age was grouped into at or over 18 months and under 18 months. The level of serum NSE was divided into at or over 100 ng/mL and under 100 ng/mL [4, 12]. Serum LDH level was divided into at or over 1400 U/L and under 1400 U/L [13]. The level of serum Tchol was defined as the high level (≥ 5.18 mmol/L) and the low level (< 5.18 mmol/L), respectively [14, 15]. Detection of MYCN amplification in tumor tissue by fluorescence in situ hybridisation. The copy of MYCN gene, namely defined as amplification, exceeded ten [16]. BMI is calculated by weight / height^2^. In combination with the other biomarkers to form a composite score, we regrouped the patients with RFS or OS based on a composite score of good (both biomarkers within the normal range), poor (neither biomarker within the normal range), and intermediate (only one biomarker within the normal range). Complete remission (CR) was denoted as the absence of all primary and metastatic lesions and the back of catecholamines and metabolites to normal levels after completing the course of treatments. RFS was defined as the appearance of new lesions after reaching CR after the treatment course. OS was defined as death from any cause after diagnosis NB.

### Follow-up

The relevant blood tests, including NSE, LDH, and Tchol in serum, were performed during each chemotherapy cycle. Imaging examinations, including magnetic resonance imaging (MRI), computed tomography (CT) and ultrasonography of the primary site and relevant metastatic sites, were rechecked every 2 chemotherapy cycles. Patients with BM metastases underwent routine BM examination, MYCN status, and minimal residual disease (MRD) examinations every 2 cycles of chemotherapy. All children underwent regular blood tests such as serum NSE, LDH, and Tchol levels, whole-body imaging examinations or positron emission tomography-CT (PET-CT) after the completion of chemotherapy.

The basic procedure for follow-up after the end of the course of treatment is (1) in the first year after the end of the course of treatment, serum NSE level and examination of the primary site (ultrasonography for the abdomen or X-ray for the chest) will be performed every 1 month, imaging evaluation will be performed every 3 months, and BM examination will be performed every 6 months for children with BM metastases at initial diagnosis. (2) In the second year after the end of the course of treatment, serum NSE level and examination of the primary site will be performed every 2 months, imaging evaluation will be performed every 4 months, and BM examination is the same as the first year after the end of the course of treatment. (3) In the third year after the end of the course of treatment, serum NSE level and examination of the primary site will be performed every 3 months, imaging evaluation will be performed every 6 months, and BM examination will be performed every 1 year for children with BM metastasis at the time of initial diagnosis. If the child suffers generalized pain and discomfort, body masses, or sudden weight loss without obvious causes, the imaging examination should be performed in time.

### Statistical methods

**Fig. 1:**
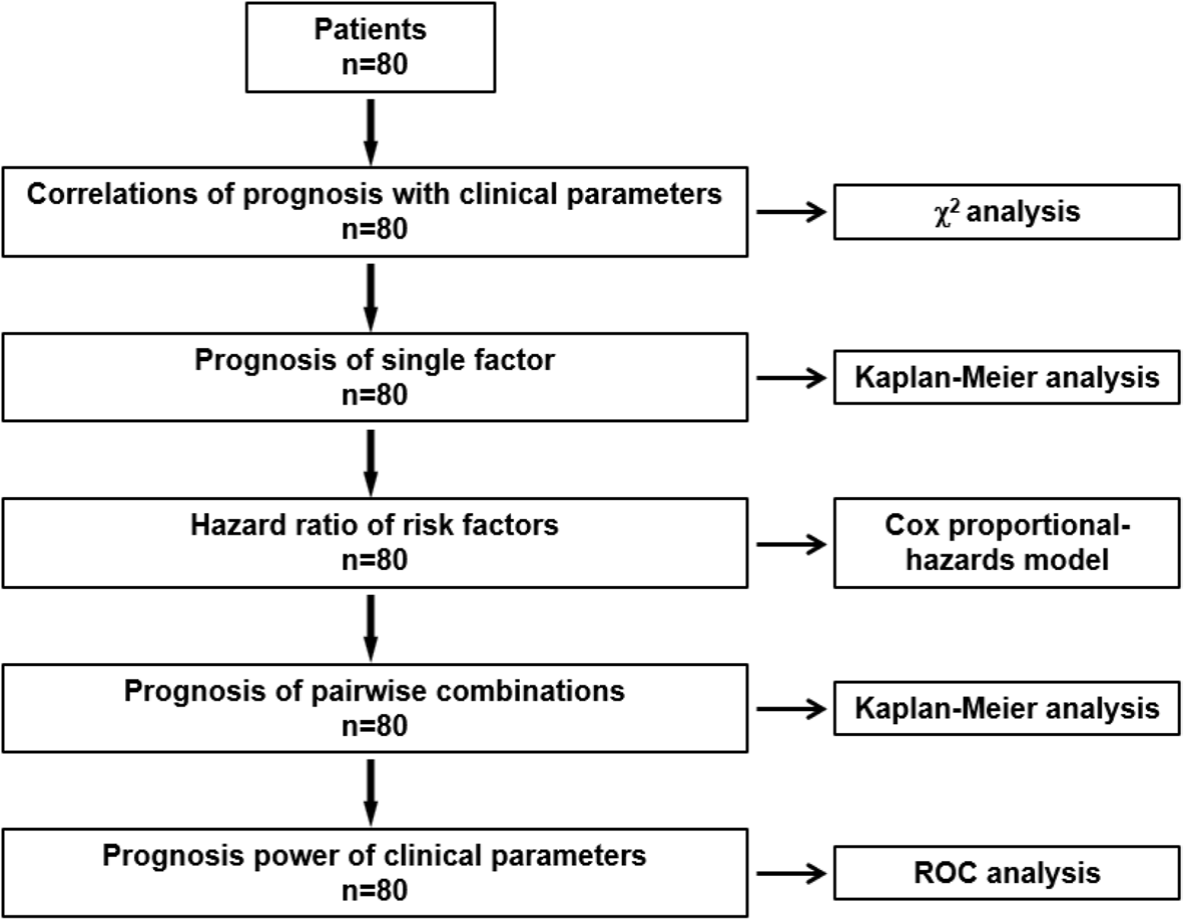
Study protocol

Statistical analysis is performed with SPSS 25.0 and Graphpad 8.0.1 software. Continuous variables with normal distribution were expressed as mean ± standard deviation. Continuous variables with non-normal distribution were expressed as median (interquartile range). Continuous variables were compared using the independent samples t-tests. The categorical variables expressed by frequency were compared using Chi-Squared test or Fisher exact test. RFS and OS were estimated by the Kaplan–Meier method and compared with the log-rank test. Multivariate analyses using the Cox proportional hazards model were performed (except for serum LDH, NSE levels and MYCN status) to identify the factors independently associated with prognosis in NB. The process in collection and analysis is shown in Fig. 1. *P* < 0.05 indicated that the difference is statistically significant.

## Result

### Basic situation

The mean age of the patients was 30.00 (12.25, 54.50) months. There were 18.75% relapsed patients and 16.25% died patients with an average follow-up time of 20.04 ± 12.63 and 17.67 (6.96, 40.48) months in this study, respectively (Supplementary Table 1). According to COG risk classification system, the children were divided into low-risk (*n* = 31), intermediate-risk (*n* = 10), and high-risk groups (*n* = 39). These three groups had significant differences in age, primary site, MYCN status, and serum levels of NSE, LDH, and Tchol (*P* < 0.05, data not shown in this article). However, other factors, such as gender, histological type and BMI, showed no significant difference in the three groups (*P* > 0.05, data not shown in this article).

### The correlation between the serum Tchol and the prognosis of the patients

**Fig. 2:**
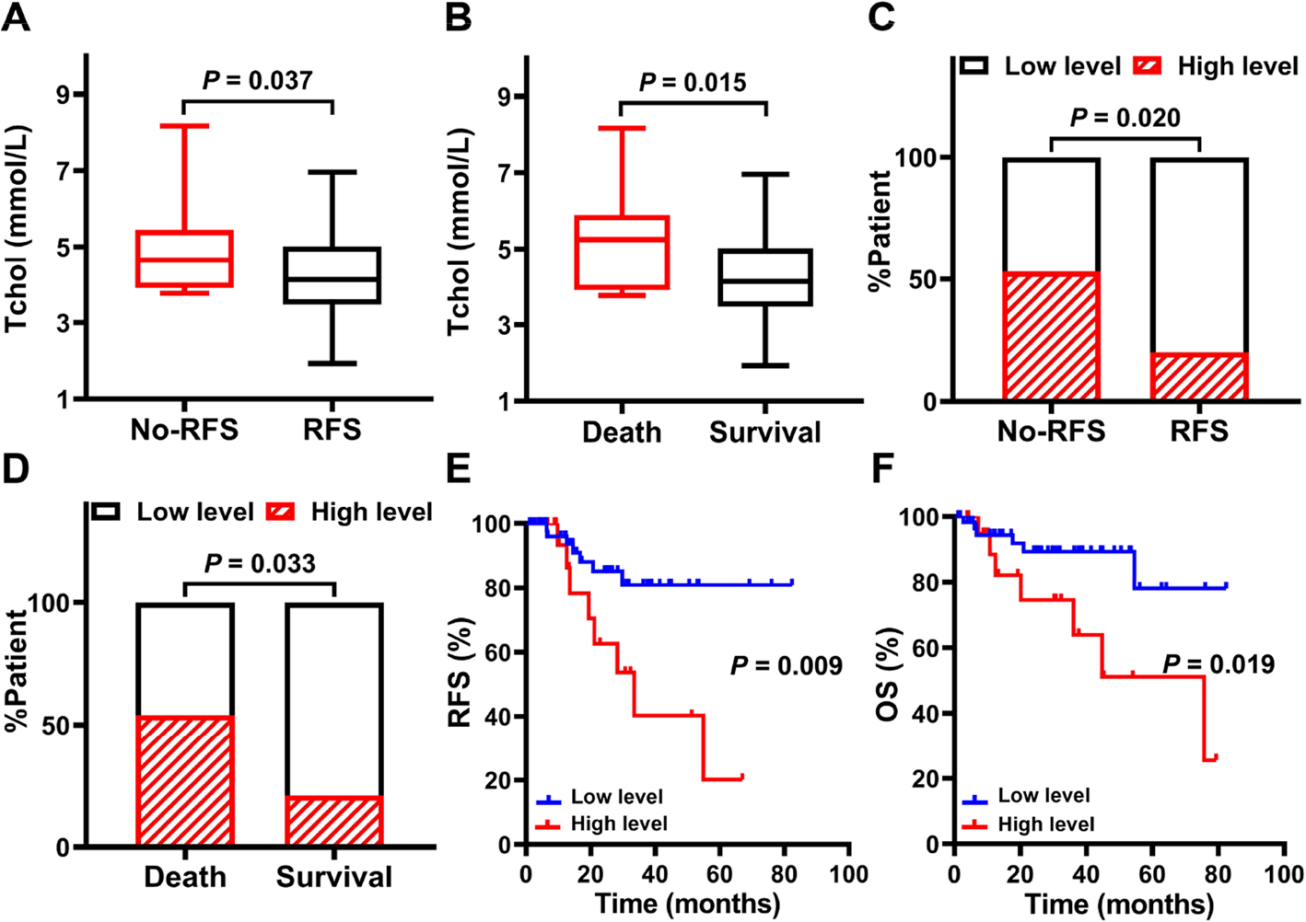
The correlation between the serum Tchol and the prognosis of the patients. **A **& **B** Serum Tchol level between the relapsed (**A**) and the died (**B**) patients. **C** & **D** The proportion of the serum Tchol between the relapsed (**C**) and the died (**D**) patients. **E** & **F** Comparison of RFS (**E**) and OS (**F**) in the groups with different serum Tchol level. Data is presented as means ± SEM. Statistical significance was calculated by independent samples t-tests and Chi-Squared test. The data was analyzed by Kaplan–Meier method. The curves were compared by Log-rank test

Next, the relationship between the serum Tchol and prognosis was studied in detail. It was found that the serum Tchol concentration was significantly higher in the outcome of the relapsed or died patients (*P* < 0.05, Fig. 2A and B). Furthermore, the percentage of the relapsed or died patients with the high level of serum Tchol was significantly higher than the patients with the low level of serum Tchol (*P* < 0.05, Fig. 2C and D). Therefore, the serum Tchol level was marked related to the prognosis of the children with NB.

### Comparison of RFS and OS in the groups

Based on the above results, we then analyzed the potential risk factors, such as age, gender, BMI, INSS stage, histological type, primary site, metastatic, serum levels of NSE, LDH, and Tchol, and MYCN status, to study the effect of prognosis. It was found that both RFS and OS in the patients with the high level of serum Tchol were significantly lower than those with the low level of serum Tchol (*P* < 0.05, Fig. 2E and F). In addition, there were significant differences in RFS between the two groups in these factors, including the age, COG risk classification system, INSS stage, primary site, BM metastasis, and serum levels of NSE, LDH (*P* < 0.05, Supplementary Fig. 2). However, the RFS was not statistically different among gender, histological types and MYCN status (*P* > 0.05, Supplementary Fig. 2).

Furthermore, there were remarkable differences in OS between the two groups in INSS stage, primary site, BM metastasis, MYCN status and serum LDH level (*P* < 0.05, Supplementary Fig. 3). However, there were no statistical differences in OS among COG risk classification system, age, gender, serum NSE level, and histological type (*P* > 0.05, Supplementary Fig. 3).

### Multivariate analysis of risk factors for recurrence and death

Next, risk factors for the results were analyzed using the COX regression model. It was found in Table 1 that the independent risk factors of RFS were INSS stage 4, and the high level of serum Tchol. The hazard ratios of INSS stage 4, and the high level of serum Tchol were 74.242 and 3.377, respectively. There was no significant effect of age, gender, BMI, primary site, histological type, or metastasis on recurrence. Moreover, it was exhibited in Table 2 that the independent risk factor of OS was only the high level of serum Tchol. The hazard ratio of the high level of serum Tchol was 4.291. Consequently, in the patients with NB, the high level of serum Tchol was a risk factor for death and recurrence.

**Table 1:**
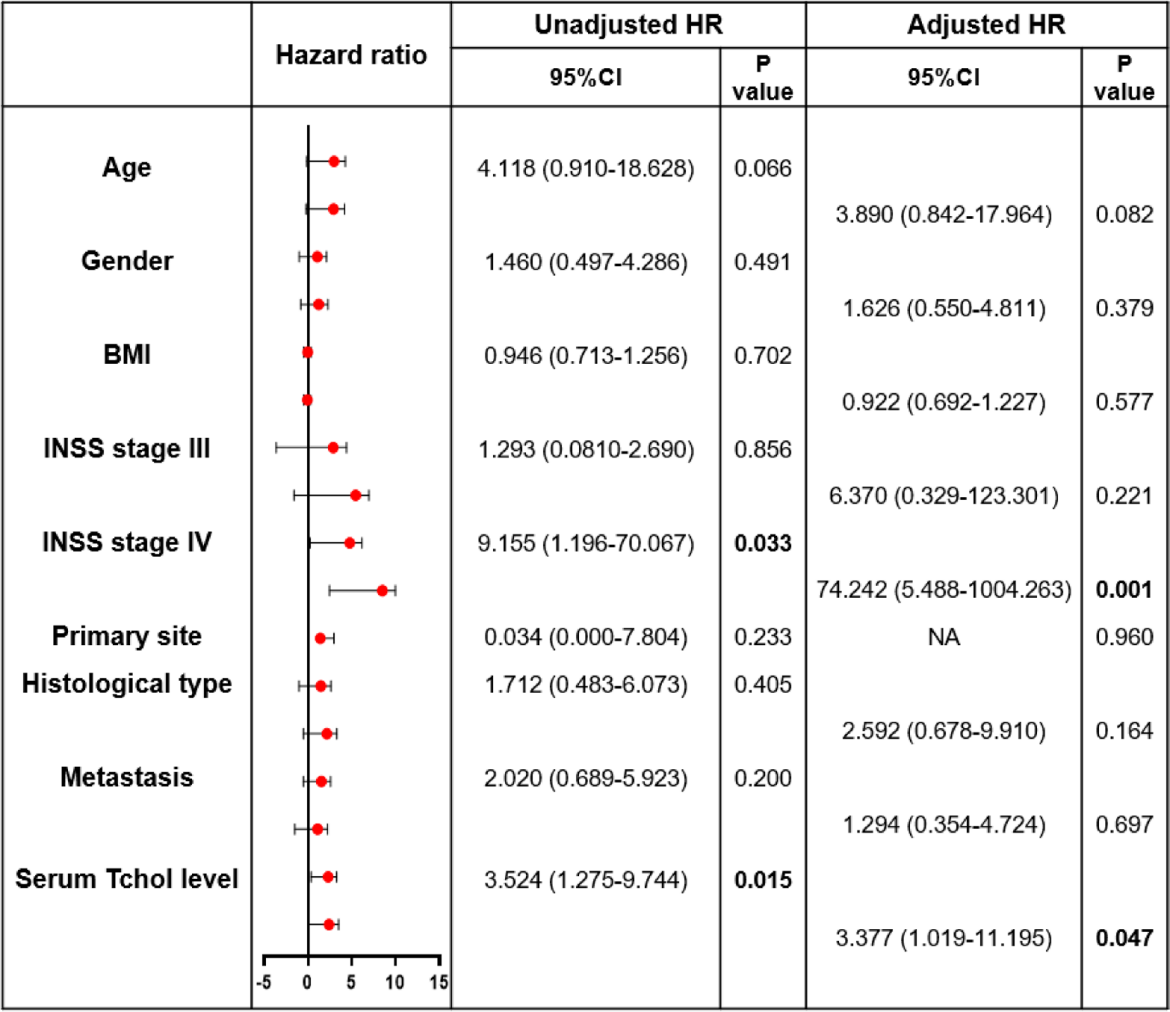
Cox proportional hazard model for the unadjusted and adjusted risk factors of recurrence with Neuroblastoma

Hazard Ratio abscissa is log base. *NA *not applicable

**Table 2:**
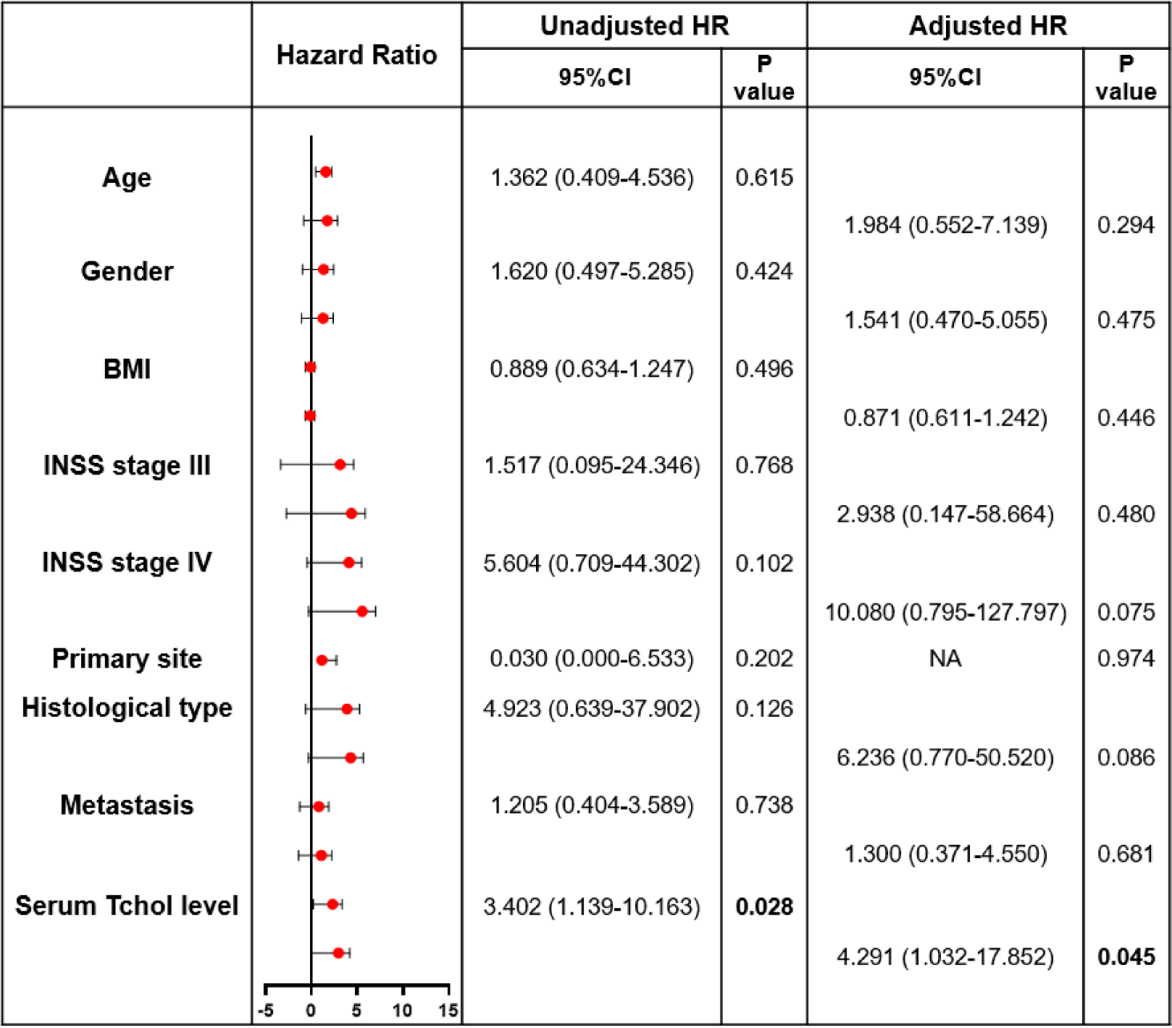
Cox proportional hazard model for the unadjusted and adjusted risk factors of death with Neuroblastoma

Hazard Ratio abscissa is log base. *NA *not applicable

### Serum Tchol level combined with other biomarkers

**Fig. 3:**
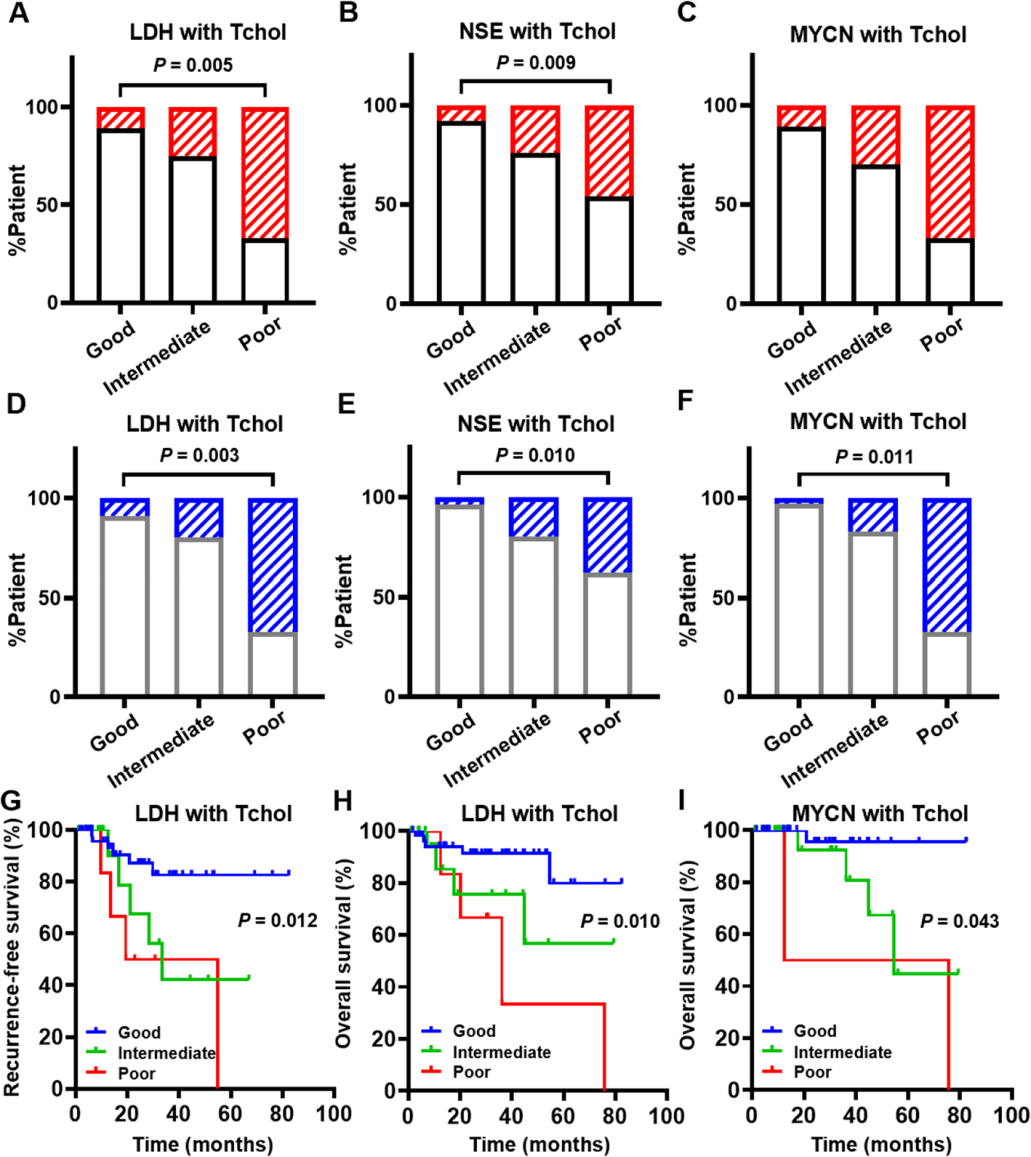
Serum Tchol level combined with other biomarkers to form a composite score. **A**-**C** In recurrence-free survival as the outcome, the proportion of the serum Tchol level combined with serum LDH (**A**), NSE (**B**) and MYCN status (**C**) in the composite score. Red twill and white without twill represent the proportion of relapsed and non-relapsed patients in the total population, respectively. **D**-**F** In overall survival as the outcome, the proportion of the serum Tchol level combined with serum LDH (**D**), NSE (**E**) and MYCN status (**F**) in the composite score. Blue twill and white without twill represent the proportion of surviving and died patients in the total population, respectively. **G** Comparison of recurrence-free survival by serum LDH and Tchol levels in the composite score. **H** & **I** Comparison of overall survival by serum Tchol level combined with the serum LDH level (**H**) and MYCN status (**I**) in the composite score. The number of patients with recurrence-free survival or overall survival was regrouped based on a composite score of good (both biomarkers within the normal range), poor (neither biomarker within the normal range), and intermediate (only one biomarker within the normal range). The data was analyzed by Kaplan–Meier method. The curves were compared by Log-rank test

We evaluated our population based on a composite prognostic score using serum Tchol levels in combination with other biomarkers, including LDH, NSE and MYCN status. Given to the score, the outcomes were differentiated into the good, the intermediate and the poor composite score groups. In the group of serum Tchol in combination with LDH, there were fewer relapsed patients in the good group (11%) compared with 30% in the intermediate group and 67% in the poor group (Fig. 3A). The number of patients who died in the good group (9%) was less than the number of patients in the intermediate group (20%) and the poor group (67%) (Fig. 3D). Besides, in the group of serum Tchol in combination with NSE, there were fewer patients with recurrence/death in the good group (8%/4%) compared with 24%/20% in the intermediate group and 46%/38% in the poor group (Fig. 3B and E). Meanwhile, in the group of serum Tchol in combination with MYCN status, there were fewer patients with recurrence/death in the good group (11%/3%) compared with 30%/17% in the intermediate group and 67%/67% in the poor group (Fig. 3C and F). Furthermore, it was shown that the RFS in the poor group was significantly lower than in the good group (*P* < 0.05, Fig. 3G). Importantly, the OS in the poor group was obviously lower than in the good group (*P* < 0.05, Fig. 3H and I). It was also exhibited that the RFS and OS in the poor group were lower than in the good group in other biomarkers combined with the serum Tchol level.

### The serum Tchol level increases the accuracy of INSS stage and COG risk classification system

**Fig. 4:**
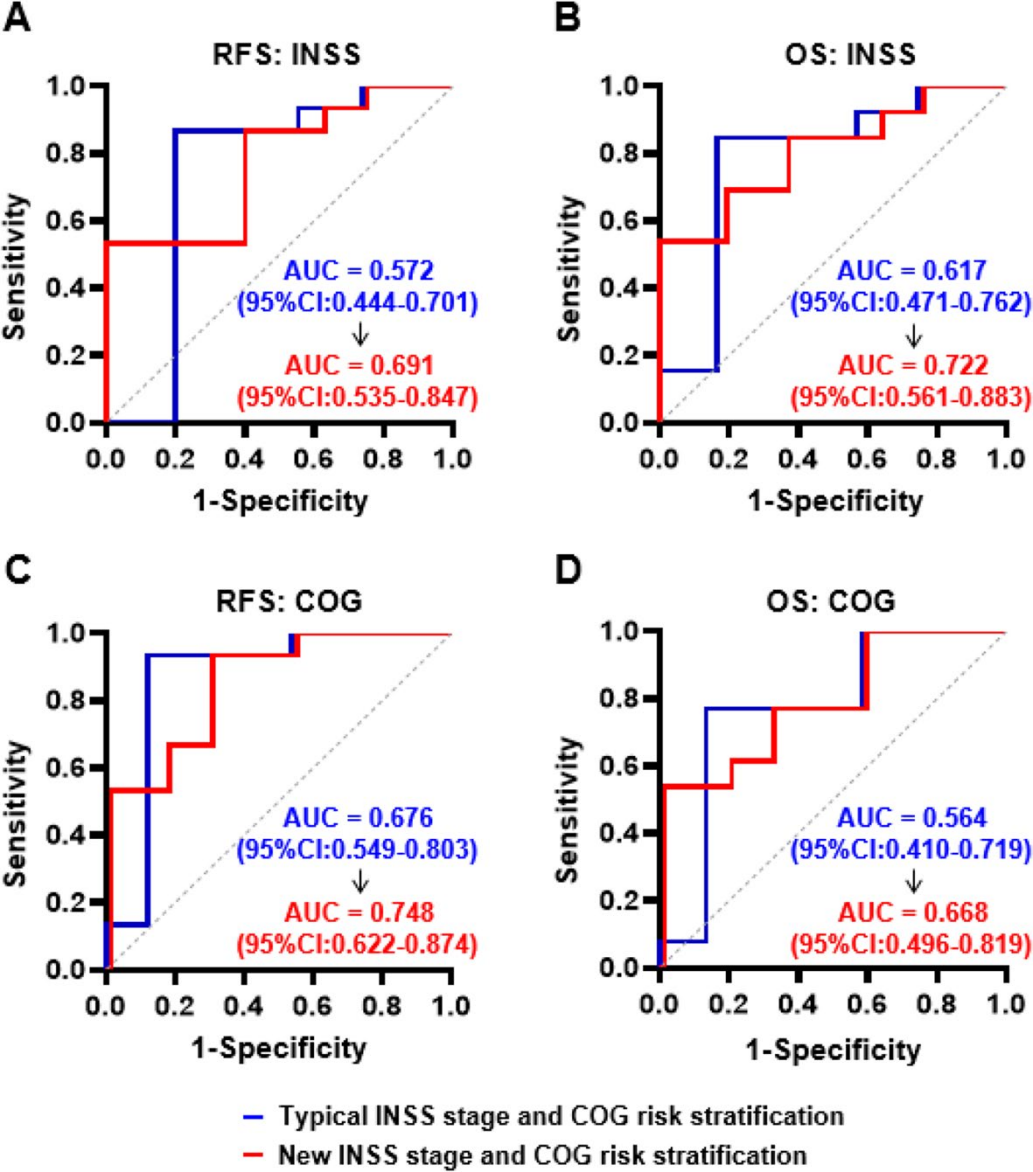
Serum Tchol level increased the accuracy of INSS stage and COG risk classification system. **A** & **B** Comparison of ROC curves with the typical and new INSS stage between the relapsed (**A**) and died (**B**) patients. **C **& **D** Comparison of ROC curves with the typical and new COG risk classification system between the relapsed (**C**) and died (**D**) patients. The data was analyzed by ROC curves. The curves were compared by the area under the curve

To investigate the impact of serum Tchol level on prognostic accuracy, the serum Tchol level as an independent factor was added into the traditional stage and risk stratification to form a new INSS stage and COG risk classification system (Fig. 4). It was found that the addition of the serum Tchol level could significantly increase the areas under the ROC curve (AUC) of INSS stage and COG risk classification system (Fig. 4A-D), indicating the increased prognostic accuracy. For RFS, the AUC for both the new INSS stage and COG risk classification system increased from 0.572 (95%CI: 0.444–0.701) to 0.691 (95%CI: 0.535–0.847) and 0.676 (95%CI: 0.549–0.803) to 0.748 (95%CI: 0.622–0.874), respectively (Fig. 4A and C). For OS, the AUC for both the new INSS stage and COG risk classification system increased from 0.617 (95%CI: 0.471–0.762) to 0.722 (95%CI: 0.561–0.883) and 0.564 (95%CI: 0.410–0.719) to 0.668 (95%CI: 0.496–0.819) (Fig. 4B and D). Therefore, the addition of serum Tchol level could significantly improve the prognostic accuracy of the INSS stage and COG risk classification system.

## Discussion

NB, a kind of tumor originating from the adrenal medulla or paravertebral sympathetic nervous system, has a variety of clinical features induced by the heterogeneity of the tumor. It was found that some infants and younger patients can spontaneously regress without any treatment or only be treated with surgery. However, most patients require further treatments, such as chemotherapy and radiotherapy [17–20]. The children with NB are usually divided into low-risk, intermediate-risk, and high-risk groups according to the INSS stage and COG risk classification system, and subsequently receive different chemotherapy regimens in clinic. However, the prognosis of the patients with classified treatments is still affected by many facts, such as age, INSS stage and MYCN status. The prognosis of the children who were diagnosed before 18 months, lower INSS stage (e.g., stages 1, 2, 4S) and MYCN non-amplification were better than those diagnosed after 18 months, higher INSS stage (e.g., stages 3 and 4) and MYCN amplification. It was also found that the primary site located in the adrenal gland was associated with poorer survival in the patients [21]. Consistent with the results of previous studies, it was exhibited in this study that these factors, including age, primary site, INSS stage, serum LDH and NSE levels, could be closely related to recurrence. Furthermore, the hazard factors, including MYCN status, INSS stage, primary site, and serum LDH level could significantly affect the survival time of patients.

Compared to the other indicators for the prognosis of patients, it is relatively expensive to test MYCN status or relative genes. However, the blood biomarkers, including NSE and LDH, have limitations with low sensitivity to predict the progression of the patients. Moreover, our results proved that the level of serum NSE could not be used to predict the survival of the patients. On the other hand, the widespread expression of LDH caused the non-specificity distribution in vivo. Therefore, it is important to find a specific biomarker for accurate prediction of outcome.

Cholesterol, a kind of lipid component, is crucial to growth and development in children. It has been found that the imbalance of cholesterol homeostasis plays a crucial role in the occurrence and progression of tumors [5, 8, 22]. For example, some studies have reported that cholesterol could cause epigenetic changes in tumors, regulate myeloid cells and promote tumor progression [5, 23]. On the other hand, cholesterol could also inhibit the in vivo growth of the liver tumor. Therefore, cholesterol has different functions in different types of tumors. It has been found that the related genes of the biosynthesis of cholesterol were up-regulated in NB [24]. Furthermore, it has been reported that the over-activation of the pathway in cholesterol biosynthesis is essential for maintaining the survival and growth of NB [25]. However, the relevant clinical studies are still lacking. Furthermore, there is unknown about the relationship between cholesterol homeostasis and the progression of NB, especially the prognosis of patients. Therefore, the relationship between cholesterol and the outcome of the patients was studied in detail. Our results demonstrated that the high level of serum Tchol was a risk factor for recurrence and death in NB. Subsequently, it was proved that the serum Tchol level as a biomarker can accurately predict the prognosis of the children with NB. In addition, we combined the serum Tchol level with the INSS stage and COG risk classification system, which are commonly used in clinical practice. It was exhibited that the addition of serum Tchol level could significantly improve the accuracy of INSS stage and COG risk classification system to predict the prognosis of NB. On the other hand, the level of serum Tchol is easier to obtain and simpler to test than other blood markers. Therefore, serum Tchol provides a new, simple, and reliable biomarker for clinical prediction of the prognosis of the children with NB. Although the clinical therapeutics have been improved, more than 50% of high-risk NB children is still lack effective treatment [26]. It has been shown that down-regulation of cholesterol not only increased tumor radiosensitivity and chemosensitivity but also improved the treatment of recurrent tumors to reduce distant metastases [27–31]. Furthermore, more and more studies have found that lower cholesterol in the tumor could improve anti-tumor therapy by regulating the tumor microenvironment [5, 32]. Although it is unknown that the mechanism of cholesterol to regulate the progression of NB, it is believed that there is a certain correlation between cholesterol and the treatment of NB, especially in patients with recurrence.

In conclusion, this study found that serum Tchol level as a simple and effective biomarker can accurately predict the prognosis of NB. It is believed that the therapeutic strategy based on cholesterol can provide a new kind of treatment for children with NB.

## Conclusion

In this study, the prognosis of children with NB was found to have a strong association with the serum Tchol level. The high level of serum Tchol is a risk factor for recurrence and death among the children with NB. The serum Tchol level as a biomarker could significantly increase the accuracy of the prediction for NB prognosis.

### Supplementary Information

**Supplementary Material 1.**

## Data Availability

The datasets generated during and/or analysed during the current study are available from the corresponding author on reasonable request. All procedures were approved by the medical ethics committee of Children's Hospital of Soochow University (application number: 2022CS132). All methods were carried out in accordance with relevant guidelines and regulations. The parents were informed of the project aims and requirements. We obtained written informed consent from the parents prior to enrollment.
