# Supplementary material for: Serum cholesterol level as a predictive biomarker for prognosis of Neuroblastoma
Source: BMC Pediatr. 2024 Mar 22;24:205. doi: 10.1186/s12887-024-04700-7 (PMC10958969; doi:10.1186/s12887-024-04700-7)
Supplement: Supplementary file 1 — Supplementary Material 1. [file 12887_2024_4700_MOESM1_ESM.docx]

**Supporting Information**

**Serum Cholesterol Level as A Predictive Biomarker for Prognosis of Neuroblastoma**

Jie Min^1+^, Yi Wu^1+^, Shungen Huang^1+^, Yanhong Li^1^, Xinjing Lv^1^, Ruze Tang^1^, He Zhao^1*^, Jian Wang^1*^

1 Children’s Hospital of Soochow University, Pediatric Research Institute of Soochow University, Suzhou, Jiangsu 215123, China

*E-mail addresses: zh2021@suda.edu.cn, jianwangsoochow@126.com

+These authors contributed equally to this work.

Supplementary Table 1: Summary of clinical characteristics

|  | **Number of cases/Sample condition** |
| --- | --- |
|  |  |
| **Age (months)** |  |
| < 18 | 27 |
| ≥18 | 53 |
| **Gender** |  |
| Male | 48 |
| Female | 32 |
| **BMI** | (14.01, 16.23) |
| **Primary site** |  |
| Adrenal glands/ Retroperitoneum | 64 |
| Others | 16 |
| **Histological type** |  |
| Unfavorable histology | 58 |
| Favorable histology | 22 |
| **Serum NSE level (ng/ml)** |  |
| < 100 | 32 |
| ≥100 | 32 |
| **MYCN status** |  |
| Unamplified | 51 |
| Amplified | 11 |
| **Serum LDH level (U/L)** |  |
| < 1400 | 69 |
| ≥1400 | 11 |
| **Serum TCHOL level (mmol/L)** |  |
| < 5.18 | 59 |
| ≥5.18 | 21 |


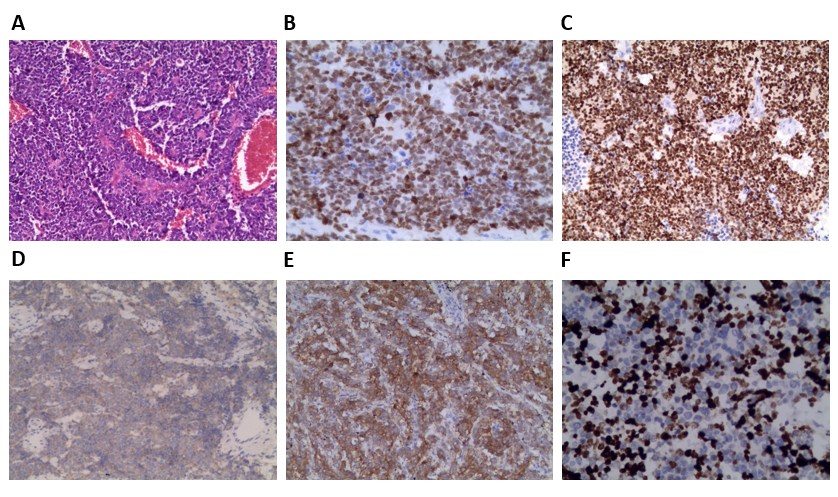


Supplementary Figure 1: (A) Hematoxylin and Eosin (H&E) staining of NB. (B-F) Representative IHC images of PHOX2B (B), GATA-3 (C), SYN (D), NSE(E) and Ki-67 (F) in NB.


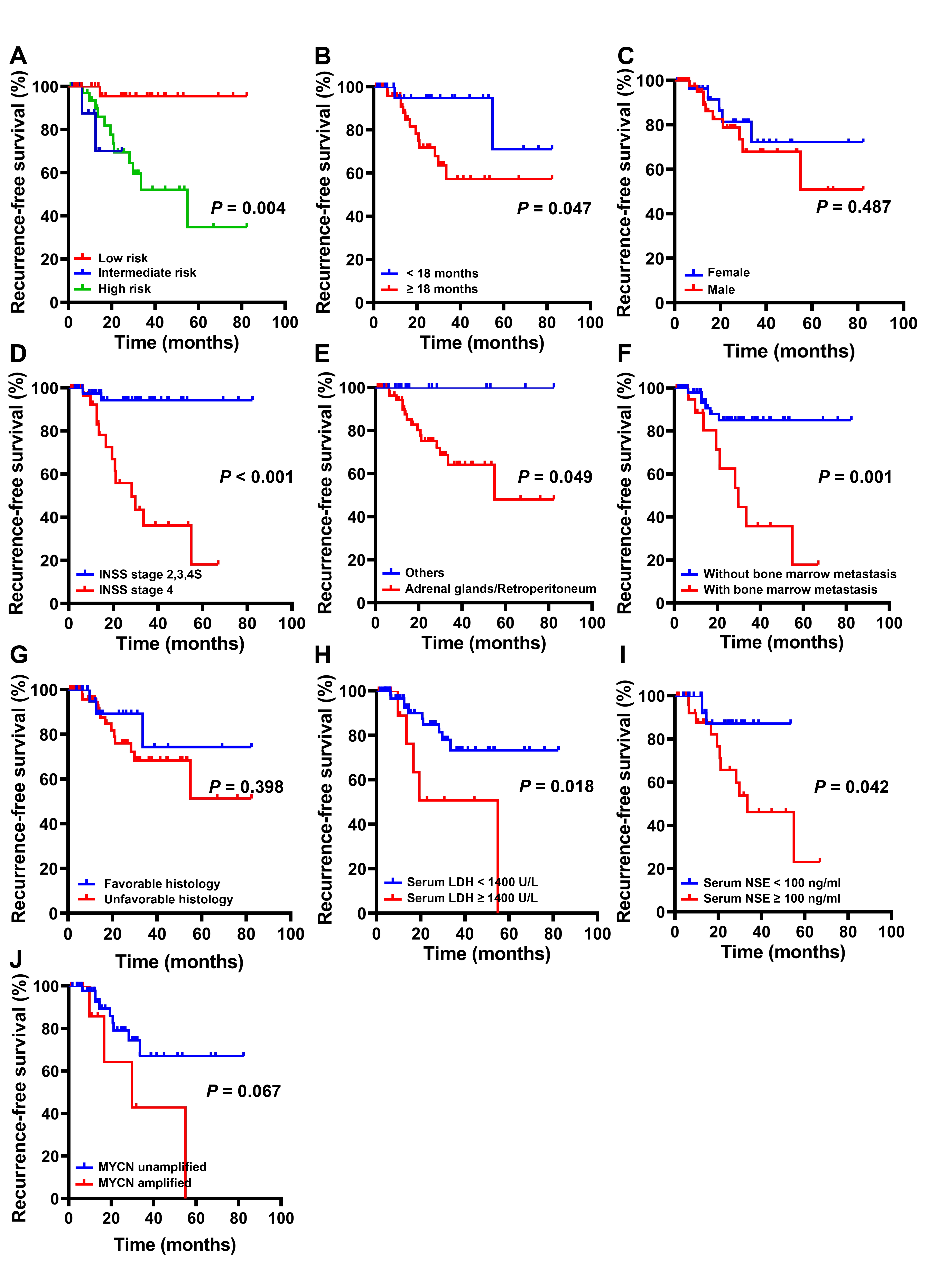


Supplementary Figure 2: Comparison of recurrence-free survival in the groups. (A-J) Comparisons of recurrence-free survival by COG risk groups (A), age (B), gender (C), INSS stage (D), primary site (E), bone marrow metastasis (F), histological type (G), serum LDH (H) and NSE (I) levels and MYCN status (J) in Neuroblastoma. The data was analyzed by Kaplan–Meier method. The curves were compared by Log-rank test.


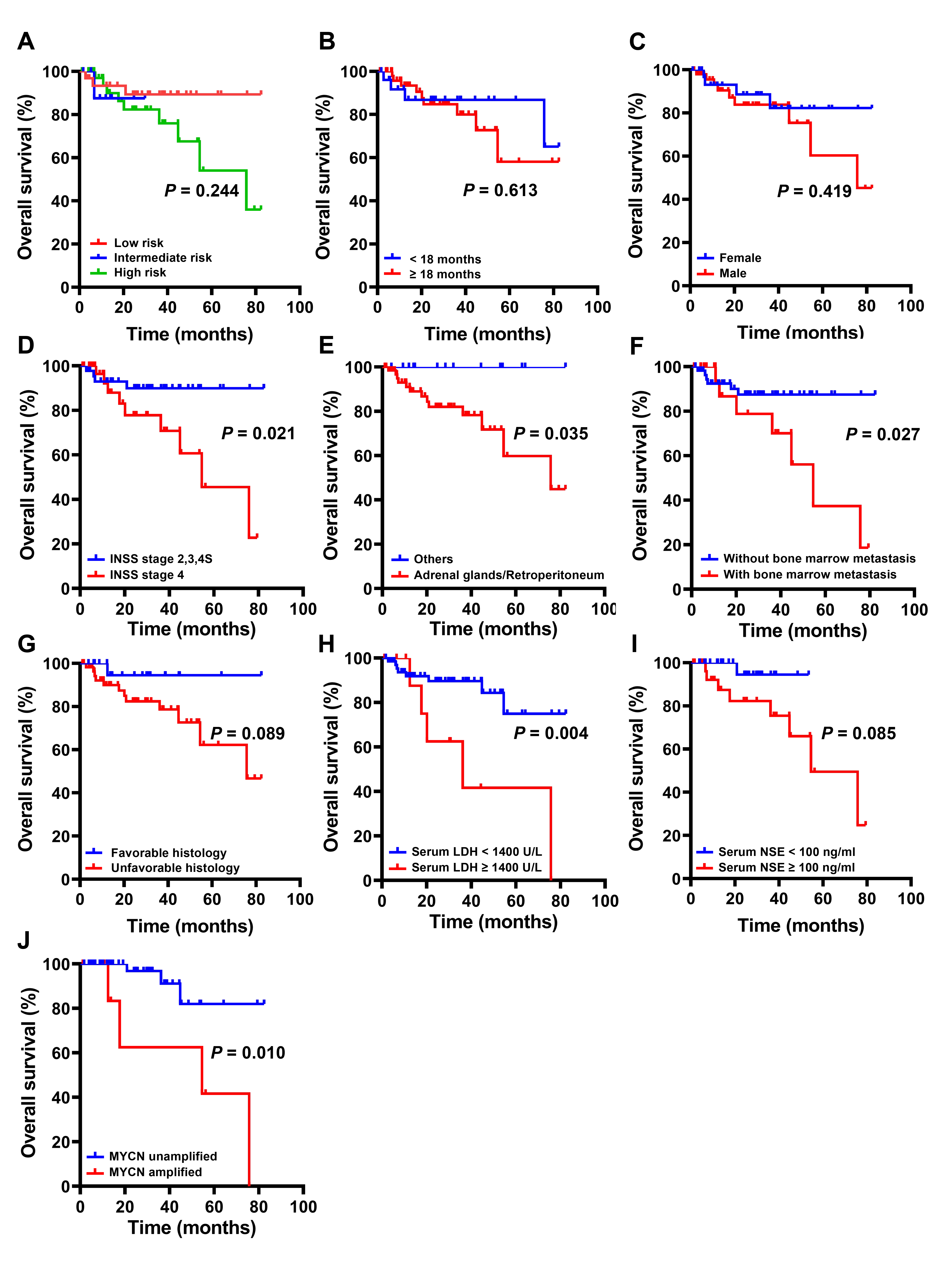


Supplementary Figure 3: Comparison of overall survival in the groups. (A-J) Comparison of overall survival by COG risk groups (A), age (B), gender (C), INSS stage (D), primary site (E), bone marrow metastasis (F), histological type (G), serum LDH (H) and NSE (I) levels and MYCN status (J) in Neuroblastoma. The data was analyzed by Kaplan–Meier method. The curves were compared by Log-rank test.
